# TOP2B is required for compartment strength changes upon retinoic acid treatment in SH-SY5Y cells

**DOI:** 10.1007/s10577-025-09764-4

**Published:** 2025-04-04

**Authors:** Erica M. Hildebrand, Ian G. Cowell, Mushtaq M. Khazeem, Snehal Sambare, Ozgun Uyan, Job Dekker, Caroline A. Austin

**Affiliations:** 1 Department of Systems Biology, University of Massachusetts Chan Medical School, Worcester, MA USA; 2 Biosciences Institute, Newcastle University, Newcastle Upon Tyne, NE2 4HH U.K.; 3 National Center of Hematology, University of Mustansiriyah, Baghdad, Baghdad, IQ Iraq; 4 Howard Hughes Medical Institute, Chevy Chase, MD USA

**Keywords:** Topoisomerase, TOP2, TOP2B, Hi-C, TAD, Chromosome compartment

## Abstract

DNA topoisomerase II beta (TOP2B) is required for correct execution of certain developmental transcriptional programs and for signal-induced transcriptional activation, including transcriptional activation by nuclear hormone ligands such as retinoic acid. In addition, TOP2B is enriched at genomic locations occupied by CCCTC-Binding factor (CTCF) and cohesin (RAD21). suggesting a role in chromosome looping and/or establishing or maintaining aspects of chromosome 3D structure. This led us to investigate the effect of TOP2B inactivation on patterns of intra- and inter- chromosomal interaction that reflect the 3D architecture of the genome. Using the retinoic acid responsive SH-SY5Y neuroblastoma cell line model, we had previously demonstrated many gene expression changes upon retinoic acid treatment and upon deletion of TOP2B. We report here that these expression changes in TOP2B null versus WT cells are accompanied by surprisingly subtle changes in local chromosome organization. However, we do observe quantitative changes in chromosome organization on a megabase scale. First, lack of TOP2B did affect compartment strength changes that occur upon ATRA treatment. Second, we observe an excess of very long-range interactions, reminiscent of interactions seen in mitotic cells, suggesting the possibility that in the absence of TOP2B some mitotic interactions are retained. Third, we see quantitative changes in centromere-telomere interactions, again indicating global changes at the megabase and chromosome level. These data support the surprising conclusion that TOP2B has only a minor role in chromosome dynamics and organization.

**Supplementary Information:**

The online version contains supplementary material available at 10.1007/s10577-025-09764-4.

## Introduction

Type II DNA topoisomerases including vertebrate TOP2A and TOP2B perform DNA decatenation and unknotting functions and modulate DNA supercoiling. These transactions occur via transient double-stranded DNA cleavage and passage of a second DNA segment through the gap before religation (Vos et al. 2011). Although enzymatically very similar, TOP2A and TOP2B differ in their physiological roles. TOP2A is expressed in proliferating cells and is essential for cell division, whereas TOP2B is expressed in both proliferating and postmitotic cells, including neurons and cardiomyocytes (Zandvliet et al. 1996, Padget et al. 2000, Tiwari et al. 2012, Zhang et al. 2012, Harkin et al. 2016, Austin et al. 2018). While TOP2B null cell lines can be grown in culture, TOP2B/Top2b nulls and hypomorphs exhibit defects in vivo in neural and B cell differentiation (Austin et al. 2018, Broderick et al. 2019), which correlates with misregulation of specific sets of genes. For example, we recently reported that loss of TOP2B expression in SH-SY5Y neuroblastoma cells resulted in altered expression of many genes, and a reduced transcriptional response to retinoic acid (RA) (Khazeem et al. 2022). Indeed, TOP2B appears to facilitate transcriptional activation in response to various stimuli including nuclear hormone receptor ligands such as estrogen, androgen, glucocorticoids and retinoic acid (Ju et al. 2006, Haffner et al. 2010, Manville et al. 2015, Trotter et al. 2015, Cowell et al. 2023), as well as neuronal activity, heat shock and growth factors (Bunch et al. 2015, Madabhushi et al. 2015). Despite these various requirements for TOP2B to maintain normal transcription programs, the precise role/s of TOP2B in these processes are unclear. Notably, topoisomerases are required to regulate negative super helical torsion that accumulates behind an elongating DNA or RNA polymerase and positive super helical torsion ahead of an elongating polymerase, as described in the twin domain model (Liu and Wang 1987; Ma and Wang 2016). Consistent with this, very long genes and genes expressed at a relatively high level were overrepresented amongst down regulated genes in TOP2B null SH-SY5Y cells (Khazeem et al. 2022). In addition, psoralen intercalation-based approaches suggest that the genome is partitioned into domains (average size ~ 100 kb) with different levels of supercoiling driven by the combination of topoisomerase and RNA polymerase activities, with negative supercoiling associated with active transcription and TOP2 chromatin binding (Naughton et al. 2013, Teves and Henikoff 2014, Kouzine et al. 2018). Using azide-trimethylpsoralen sequencing, megabase scale supercoiling domains were also observed across the genome that align with compartments. Negatively supercoiled domains aligned with A compartments and positively supercoiled domains with B compartments, these supercoiling domains were modulated by TOP1 and TOP2B (Yao et al. 2024). Furthermore, ChIP-seq analysis has revealed that peaks of TOP2B chromatin binding frequently overlap with CTCF/cohesin binding sites which can be found at the base of chromatin loops and topologically associating domains (TADs) (Uuskula-Reimand et al. 2016, Canela et al. 2017). The connection between TOP2, supercoiling and chromatin/chromosome organization described above led us to investigate the effect of TOP2B removal on patterns of intra- and inter- chromosomal interaction reflecting the 3D architecture of the genome.

Chromosomes are organized into structural domains via multiple mechanisms. TADs, which are regions which interact more within the domains than between domains along a chromosome, are formed on the scale of 10-100kb. TADs are formed through a loop extrusion mechanism, where cohesin forms DNA loops and translocates along the DNA molecule until reaching convergent CTCF sites or other barriers to loop extrusion such as active promoters (Fudenberg et al. 2016). Enhancer-promoter interactions tend to occur within TADs, although recent evidence suggests that these interactions can also occur across TAD boundaries (Hsieh et al. 2022, Zuin et al. 2022, Hung et al. 2024). TADs are observed on a Hi-C heatmap as triangles or squares along the diagonal, often with enriched looping interactions between their boundaries. Compartments, which are regions of chromatin that interact with other regions of similar regulatory or chromatin status (active-active, or inactive-inactive), can be found at multiple size ranges, both smaller and larger than TADs, and are different from TADs in that they form long-range interactions with other compartment regions both on the same chromosome and on different chromosomes (Lieberman-Aiden et al. 2009, Rao et al. 2014). Compartments may be formed by a phase separation mechanism, which condenses assemblies of molecules into specific regions of the nucleus based on biophysical properties (Erdel and Rippe 2018; Hildebrand and Dekker 2020). On a Hi-C heatmap, compartments can be observed as a checkerboard pattern (Hildebrand and Dekker 2020). There are two main compartment types, A, which contains active euchromatin, and B, which contains inactive heterochromatin. The A and B compartments can be further subdivided into sub compartments, containing specific histone marks and activation/silencing states (Rao et al. 2014; Hildebrand and Dekker 2020, Spracklin et al. 2023).

Considering the effect of TOP2B inactivation on gene expression and gene activation by factors such as RA, together with its association with CTCF, we hypothesized that TOP2B would play a role in the 3D organization of the genome at one or more scales. With this in mind, we compared Hi-C interaction patterns from wild-type (WT) and *TOP2B*^*−/−*^ (BKO) SH-SY5Y cells under control growth conditions and after exposure to RA (all-trans retinoic acid – ATRA) for 5 days to induce cell cycle arrest and differentiation. In a previous study 1141 genes were significantly upregulated and 969 downregulated by ATRA in WT cells and 1472 genes were downregulated and 1098 upregulated in BKO compared to WT cells after 24 h exposure to retinoic acid (Khazeem et al. 2022). These gene expression changes were not accompanied by changes in cohesin loop/TADs but there were changes in intrachromosomal interactions at the larger (> Mb) scale. Also, in the absence of TOP2B there were compartment strength changes that occurred upon ATRA treatment.

## Results and discussion

TOP2B is a constitutively expressed chromatin protein, intimately involved in regulating DNA supercoiling state and required for the correct execution of transcriptional programs and induction of genes by agents such as nuclear hormone receptor agonists. To investigate to what degree this reflects a role for TOP2B the 3D architecture of the nucleus we have focused on Hi-C analysis of both WT and TOP2B null sublines of the RA-responsive SH-SY5Y cell line.

### Gene expression and cell cycle profile

**Fig. 1:**
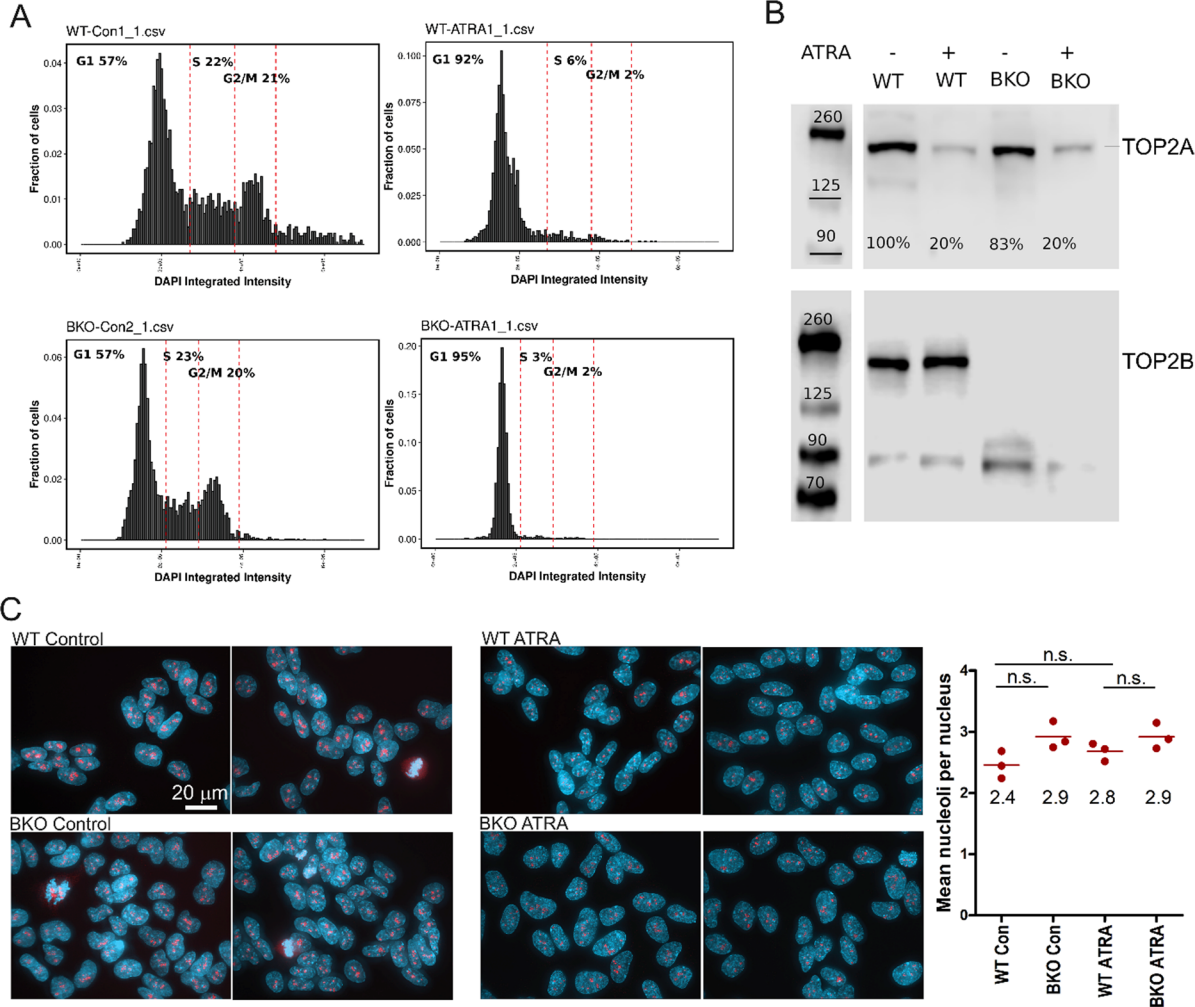
**TOP2B deletion does not affect ATRA-induced G1 arrest, ATRA-induced downregulation of TOP2A or overall nuclear morphology**. **A** For ATRA treatment WT and TOP2B^−/−^ (BKO) SH-SY5Y cells were incubated in medium containing 10µM ATRA for 5 days. Cell cycle distribution was determined using quantitative DAPI fluorescence microscopy (Roukos et al. 2015). G1, S, and G2/M gate boundaries are shown as dotted red lines. **B** Western blots illustrating ATRA-induced TOP2A downregulation and verifying null TOP2B phenotype of BKO cells (25µg whole cell extract per well). Anti-TOP2A (top) and anti-TOP2B (bottom). **C** Immunofluorescence images of WT and BKO cells grown under control conditions or after 5 days of ATRA exposure. Left—Cells were stained for fibrillarin (red) and counterstained with DAPI (blue). Right—For each condition the number of fibrillarin staining foci (nucleoli) per nucleus were manually counted from three fields of cells (at least 20 cells per field) and mean number of foci per nucleus were calculated and plotted. The mean of the means from each field obtained for each condition is indicated on the graph. Statistical significance was determined using one-way Anova with Tukey post-hoc test

RA treatment results in altered expression of many genes in SH-SY5Y cells. RNA-seq analysis previously revealed increased expression of over 1100 genes and reduced expression of nearly 700 genes after 24 h of retinoic acid treatment (Khazeem et al. 2022). This included robust induction of *CYP26A1*, *CYP26B1*, *CRABP2* and *DHRS3* that are involved in RA metabolism and transport, and of neurogenesis-associated genes such as *NTRK2*. TOP2B deletion resulted in reduced expression of almost 1500 genes and increased expression of nearly 1000 genes and blunted the transcriptional response to RA (Khazeem et al. 2022). RA treatment leads to differentiation of SH-SY5Y cells into adrenergic neuron-like cells, accompanied by cell cycle exit (Bell et al. 2013, Kovalevich and Langford 2013) and since cell cycle distribution and particularly the proportion of cells in a G1 arrested state could affect patterns of intra- and inter- chromosomal contacts we first examined the cell cycle distribution of wild type (WT) and TOP2B^−/−^ (BKO) cells before and after RA treatment. Before RA treatment WT and BKO cells exhibited very similar cell cycle profiles (Fig. 1A) with 57% of cells exhibiting G1 DNA content. After 5 days exposure to RA the proportion of G1 cells increased to over 90% in both cell lines. This was accompanied by a large reduction in the expression of TOP2A as predicted for cells exiting the cell cycle, while TOP2B expression was unaffected by ATRA treatment (Fig. 1B). The fall in TOP2A expression after RA treatment (80%) matches that observed previously by quantitative immunofluorescence (Khazeem et al. 2022). We conclude that initial cell cycle distributions, and changes in those distributions after RA treatment were similar for both cell lines.

### Nuclear morphology, nucleolar shape and number

To determine whether RA-induced differentiation or loss of TOP2B expression is associated with changes in overall nuclear morphology, including features such as nucleolar size and number, we carried out immunofluorescence analysis of WT and BKO SH-SY5Y cells using the nucleolar fibrillar center marker fibrillarin and the DNA fluorescent stain DAPI. The number of bright fibrillarin-staining foci identifying nucleoli were not significantly different comparing WT and BKO cells before and after RA treatment (Fig. 1C). We previously observed that genes downregulated in RA-treated cells were highly enriched for ribosome biogenesis GO:Biological Process terms (Khazeem et al. 2022), and consistent with this, visual inspection of images revealed that fibrillarin staining foci were smaller and less intensely stained with anti-fibrillarin in both cell lines after RA treatment.

### Inter-chromosomal interactions increase with ATRA differentiation

**Fig. 2:**
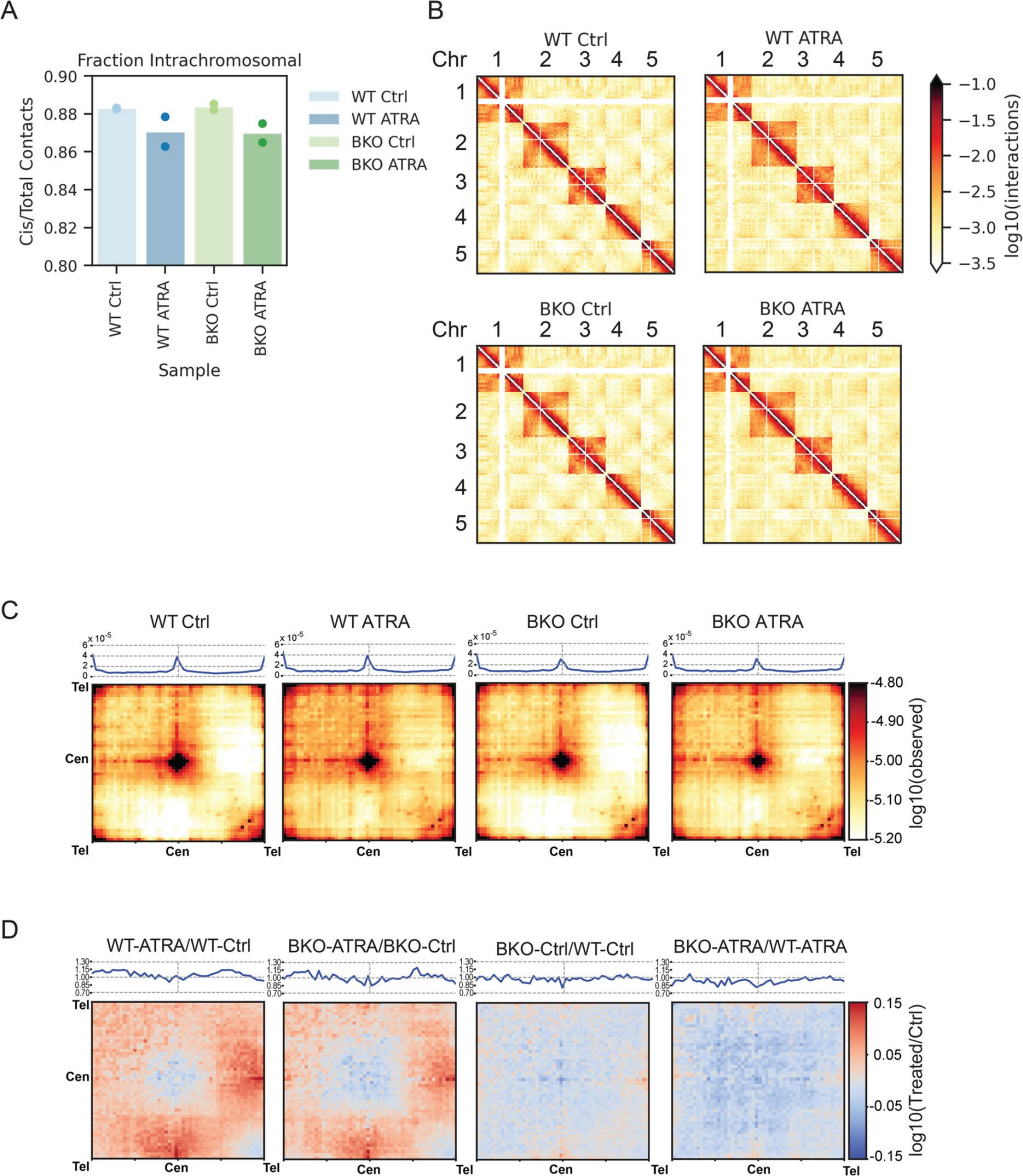
**Inter-chromosomal interactions increase with ATRA differentiation**. **A** Fraction of Hi-C interactions within chromosomes/total interactions for all chromosomes (light blue: WT Ctrl, dark blue: WT ATRA, light green: BKO Ctrl, dark green: BKO ATRA) *n* = 2. **B** Hi-C heatmap of Chr1-Chr5. Log10 (interactions) between 5Mb genomic bins are plotted for each sample. **C** Average scaled inter-chromosomal pileups as a heatmap, log10 (observed Hi-C interactions), with a line plot of the diagonal of the matrix (top left to bottom right) above each heatmap for all chromosomes (*n* = 2, combined). D. Average scaled inter-chromosomal heatmaps comparing between conditions (log10 ratio, condition pairs as indicated), with a line plot of the diagonal line (top left to bottom right) down above each heatmap for all chromosomes (*n* = 2, combined)

To explore chromosome architecture and nuclear organization we performed Hi-C (Lieberman et al. 2009). Hi-C libraries were prepared for four conditions: WT and BKO cells before and after RA treatment (5 days). The fraction of intra-chromosomal reads across all chromosomes is highest in the control conditions and is slightly decreased in the ATRA conditions. This change may be due at least in part to the change in cell cycle profiles with fewer mitotic cells upon ATRA differentiation (in both WT and BKO cells), as mitotic chromosomes have a higher intrachromosomal fraction than interphase chromosomes (Naumova et al. 2013) (Fig. 2A). Hi-C heatmaps of the intra and inter-chromosomal interactions for the 5 largest chromosomes (chr1-5) using 5Mb genomic bins show that upon ATRA treatment, the Rabl-conformation along chromosome arms (in cis) and clustering of centromeres and telomeres between chromosomes decreased, while interactions between chromosome arms in trans increased (Fig. 2B). We quantified this effect genome-wide by plotting the average scaled chromosome-chromosome interaction map (Fig. 2C). Centromere and telomere clustering is apparent in WT Ctrl and BKO Ctrl conditions, with fewer interactions between other parts of the chromosome arm, especially between the centromeres and the long arms of other chromosomes. In both ATRA treated conditions there is an increase in inter-chromosomal interactions on chromosome arms, and a decrease in centromere-centromere and telomere-telomere interactions. The reduced centromere-centromere and telomere-telomere interactions in response to ATRA are apparent in the log10 ratio heatmaps showing WT-ATRA vs WT-Ctrl and BKO-ATRA vs BKO-Ctrl (Fig. 2D). The effect of BKO is subtle. There is a small reduction in centromere-centromere interactions in the BKO-Ctrl vs WT-Ctrl, and less of an increase in inter-chromosomal arm-arm interactions in BKO-ATRA compared to WT-ATRA, but more of a decrease in cen-cen interactions (Fig. 2D). Thus, the changes in these interactions that occur in cells treated with ATRA are attenuated when *TOP2B* is deleted.

### Topologically associating domains are unchanged

**Fig. 3:**
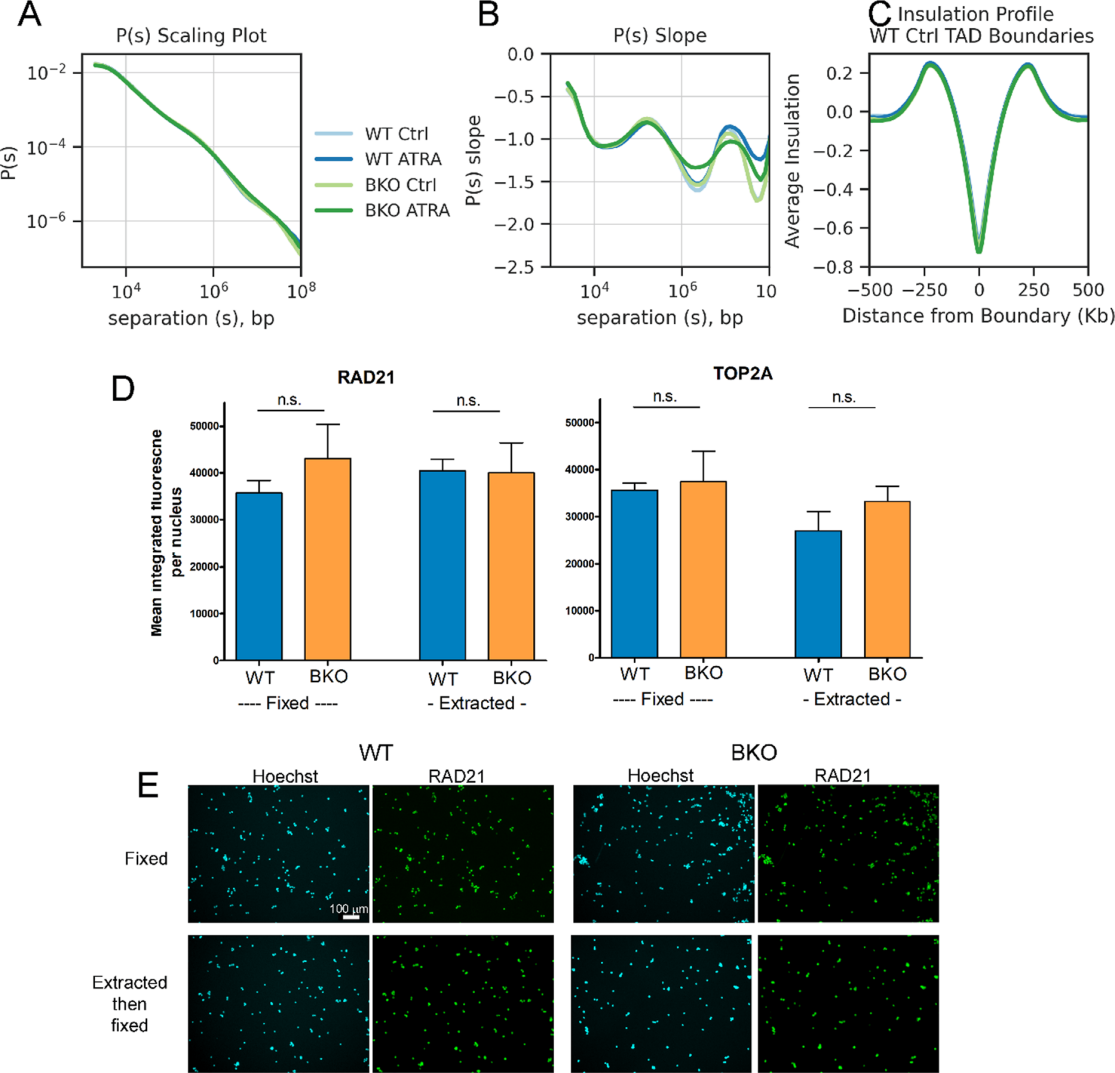
**Topologically associating domains and cohesin are unchanged in WT versus BKO cells**. **A** P(s) scaling plot showing average Hi-C interaction frequency at each distance bin for all chromosomes (light blue: WT Ctrl, dark blue: WT ATRA, light green: BKO Ctrl, dark green: BKO ATRA) (*n* = 2, combined). **B** Slope of P(s) scaling plot for each distance bin for all chromosomes (light blue: WT Ctrl, dark blue: WT ATRA, light green: BKO Ctrl, dark green: BKO ATRA) (*n* = 2, combined). **C** Average insulation profile in each condition at TAD boundaries called in the WT Control sample for all chromosomes (light blue: WT Ctrl, dark blue: WT ATRA, light green: BKO Ctrl, dark green: BKO ATRA) (*n* = 2, combined). **D** Immunofluorescent quantification of RAD21 and TOP2A in CSK1-extracted and paraformaldehyde fixed WT (blue) and BKO (orange) cells with fixation alone or with extraction then fixation. Images were captured from three replica slides per condition. Bars represent the means of the median RAD21 immunofluorescence for each replica slide ± S.D. Significance testing was by t-test (2-way unpaired). **E** Representative images of RAD21 immunofluorescence, Scale bar = 100µm

Next, we investigated how intra-chromosomal interactions were changed with ATRA differentiation in WT and BKO cells. The *P*(*s*) plot (contact probability *P* as a function of genomic distance *s*) shows average interactions binned by distance and shows a slight change at longer distances with ATRA-treated BKO cells, specifically – between 10^6 and 10^7 bp (1-10Mb) of separation (Fig. 3A). The slope of the *P*(*s*) plot highlights this change, with an increase in the slope in the dip near 10^6 for BKO + ATRA compared to the other samples, and a decrease in the slope at the peak near 10^7 bp (Fig. 3B). Similar observations were seen in an independent study with TOP2 inhibitors (Baxter 2024, Hildebrand et al. 2024) and were consistent with retention of mitotic interactions in G1. Importantly, no change was observed in the first peak (near 10^5), which corresponds to the average size of cohesin loops (Polovnikov and Slavov 2023) which contribute to formation of topologically associating domains (TADs). The average insulation profile at TAD boundaries also shows no change across the conditions, showing that there is no global change in the strength of TADs in BKO cells (Fig. 3C).

### Cohesin abundance and chromatin binding

The relative abundance and chromatin binding of cohesin RAD21 was assessed by quantitative immunofluorescence using a pre-fixation detergent buffer (CSK1)(Poonperm et al. 2017) to extract non chromatin bound RAD21 (Fig. 3D). As a control we assayed TOP2A, another chromatin associated protein. No significant difference was observed between the WT and BKO cells, either for total or chromatin bound RAD21 or TOP2A. Thus, consistent with the lack of effect on interactions at the 10^5^ bp scale, TOP2B deletion does not affect expression or chromatin association of the cohesin protein RAD21 that is required for loop domains.

### TOP2B is required for compartment strength changes upon ATRA differentiation

**Fig. 4:**
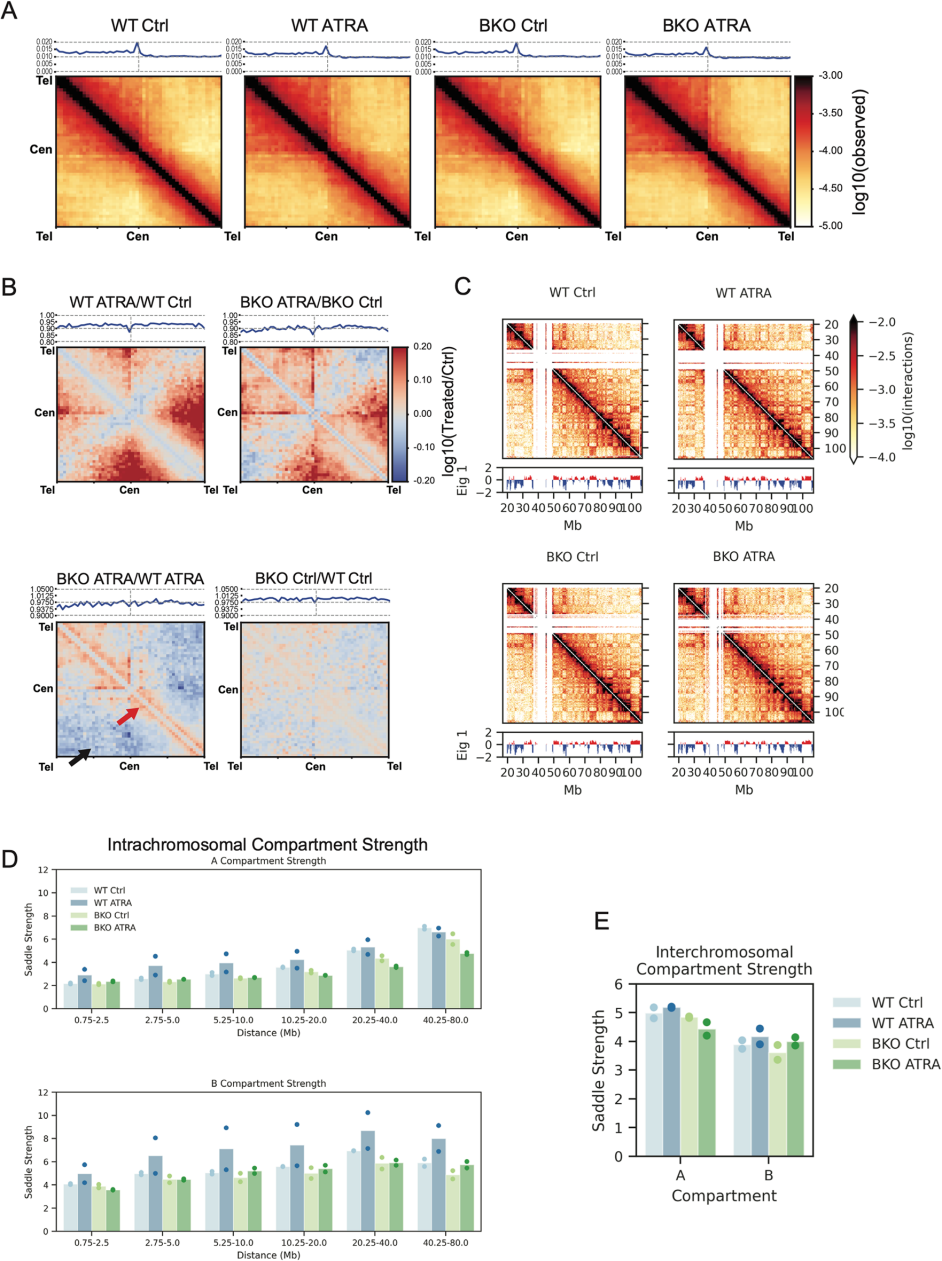
**TOP2B is required for compartment strength changes upon ATRA differentiation**. **A** Average scaled intra-chromosomal Hi-C interactions with lineplot of the diagonal of the matrix (top left to bottom right) for each sample for all chromosomes (*n* = 2, combined). **B** Comparisons between the average scaled intra-chromosomal interactions across all chromosomes for pairs of conditions as indicated, as the log10 ratio of the observed Hi-C interactions between the two conditions, with lineplot showing the diagonal of the matrix plotted above each (*n* = 2, combined). **C** Hi-C heatmap of Chr14p Log10(interactions) between 250kb genomic bins are plotted for each sample. Compartment score (Eigenvector 1) is plotted below each heatmap. A compartment (active/euchromatin) is shown in red, B compartment (inactive/heterochromatin) is shown in blue (*n* = 2, combined). **D** Intra-chromosomal compartment strength in the A or B compartment for all chromosomes, separated by genomic distance (light blue: WT Ctrl, dark blue: WT ATRA, light green: BKO Ctrl, dark green: BKO ATRA) (*n* = 2). *E*. Interchromosomal compartment strength in the A or B compartment for all chromosomes (light blue: WT Ctrl, dark blue: WT ATRA, light green: BKO Ctrl, dark green: BKO ATRA) (*n* = 2)

We next investigated the intrachromosomal interactions by plotting the average intrachromosomal interactions across all chromosomes (Fig. 4A). In both genotypes, ATRA treatment reduced interactions along both the main diagonal and perpendicular to the main diagonal, consistent with a decrease in Rabl conformation (Fig. 4B). Long-range intra-arm interactions were increased upon ATRA treatment. BKO-Ctrl vs WT-Ctrl did not show many differences, while BKO-ATRA vs WT-ATRA show a decrease in long-range chromosome arm interactions (black arrow) and an increase in interactions closer to the main diagonal (red arrow). This retention of interactions close to the diagonal suggests that BKO-ATRA cells are unable to correctly and fully transition to the non-dividing genome-folding state without TOP2B (Fig. 4B).

As the *P*(*s*) plot showed changes at the scale of 1-10Mb in the BKO + ATRA sample (Fig. 3B), this suggested that compartment interactions, which dominate *P*(*s*) at that length scale, might be affected. We examined compartmentalization by plotting Hi-C interaction maps of the q-arm of chr14 for each sample, along with the compartment profile, as reflected by the first eigenvector obtained with principal component analysis of the Hi-C interaction map (Lieberman-Aiden et al. 2009) (Fig. 4C). Although the Eigenvector plots are very similar for WT and BKO Control conditions, compartments appear sharper and more defined in the WT ATRA sample compared to the WT Control, however the change is less apparent in the BKO cell line. Quantification of intra-chromosomal compartment strength using the saddleplot quantification method (Nora et al. 2017) was performed for loci separated by different genomic distances (Fig. 4D). In the A compartment, there are changes in compartment strength rank between conditions across the distances. In WT cells, ATRA treatment results in stronger compartments at shorter distances (< 20Mb), but unchanged or slightly weaker compartments at distances longer than 20Mb. In BKO cells, there is only a slight increase in A compartment strength at shorter distances with ATRA, and there is loss in compartment strength at longer distances with ATRA (> 20Mb). This suggests that the increase in A compartment strength at shorter distances upon ATRA treatment requires TOP2B. In the B compartment, the compartment strength rank is similar across distances, and shows that while WT ATRA has the strongest B compartment strength, BKO ATRA does not show this same magnitude increase vs BKO Ctrl. Therefore, TOP2B is required for the increase in B compartment strength at all distances, upon ATRA treatment.

Between chromosomes, the A compartment strength is only slightly increased in the WT cells upon ATRA treatment, with a larger decrease in A compartment strength in BKO ATRA vs BKO WT (Fig. 4E). In the B compartment, interchromosomal compartment strength is slightly increased upon ATRA treatment in both WT and BKO cells. Only the A compartment intrachromosomal compartment interaction strength changes upon ATRA treatment require TOP2B.

To summarize, we find that the strength of compartments, which normally increases in WT cells upon ATRA differentiation, is reduced/attenuated in the BKO ATRA treated cells. There is a distance dependent effect in the A compartment, and a more pronounced difference in compartment strength between WT and BKO cells within chromosomes rather than between chromosomes. This may reflect an inability to reorganize compartments due to decreased chromatin mobility without TOP2B induced strand passage, as has been suggested for removal of inter-chromosomal entanglements (Brahmachari and Marko 2019), or could be a remnant of mitotic chromosome entanglement that is not resolved in the absence of TOP2B, thus not allowing for compartments to fully form even when cells are no longer dividing following ATRA treatment (Hildebrand et al. 2024).

### Compartment score changes at select regions are correlated with changes in gene expression

**Fig. 5:**
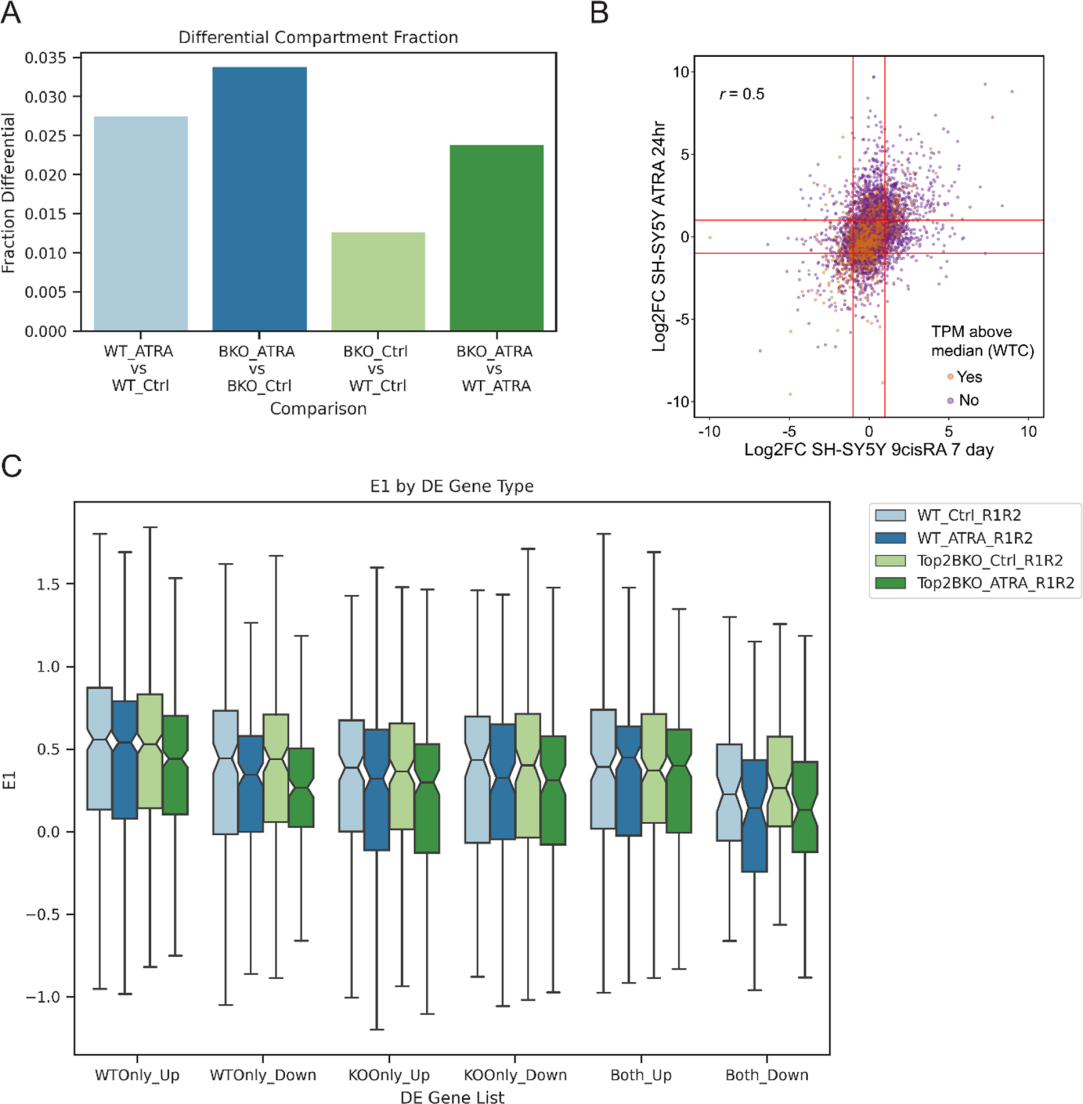
**Correlation between gene expression changes and compartment strength changes**. **A** Differential compartment fractions called using dcHiC-v1 (FDR < 0.01) in the WT or BKO cell lines upon ATRA differentiation (light blue: WT ATRA vs WT Ctrl, dark blue: BKO ATRA vs BKO Ctrl, light green: BKO Ctrl vs WT Ctrl, dark green: BKO ATRA vs WT ATRA). **B** Comparison of gene expression changes observed after 24 h exposure to 10µM ATRA versus 7 days exposure to 9cisRA, separated for more highly expressed genes (TPM > median in WT control cells, orange), and less highly expressed genes (tpm < median in WT control cells, purple). Pearson’s correlation coefficient *r*, comparing Log2 fold change values for genes at the two time points, is indicated. **C** Notched box-plot showing the distribution of eigenvector 1 compartment score in all bins overlapping differentially expressed genes (light blue: WT Ctrl, dark blue: WT ATRA, light green: BKO Ctrl, dark green: BKO ATRA), (*n* = 2, combined), separated by which comparisons the genes were differentially expressed between (WT Only, BKO Only, or Both) and direction of differential expression (Up or Down). Notches indicate 95% CI of the median

To investigate the functional correlations of the changes in compartment strength, we performed an integrated analysis comparing the Hi-C compartment results with the genomic locations of differentially expressed genes from RNA-seq datasets for 24h and 7 day RA treatment in SH-SY5Y cells (Khazeem et al. 2022). We first used the analysis package dcHiC to call differential compartment regions in the WT or BKO cell lines upon ATRA differentiation (Chakraborty et al. 2022). We found that the eigenvector of 2.75% of compartment bins were significantly changed during WT differentiation, and 3.39% were significantly changed with BKO differentiation (FDR < 0.01). In the control treatment, 1.27% of bins were significantly different between WT and BKO, while with ATRA treatment 2.39% of bins were changed between the two cell lines (FDR < 0.01) (Fig. 5A).

Comparing the two different RNA-seq data sets (24 h and 7 days RA treatment), we found that gene expression changes were moderately correlated at the two time points after RA treatment (Fig. 5B, Pearson correlation coefficient of 0.5). Therefore, we next compared the genes with their transcription start site positioned within these 250kb differential compartment bins to genes that were differentially expressed in the same direction as the compartment changes with 24 h of ATRA treatment, as this dataset includes the BKO cell line. In the aggregate data we found a significant overlap (hypergeometric distribution *p* value < 0.05) only for the following comparisons: 1. Genes that were upregulated by ATRA in the BKO cell line and genes located in A compartment regions with significant increases in the value of eigenvector 1 upon ATRA treatment in the BKO cell line (Table 1). Therefore, this set of genes in BKO cells are upregulated by ATRA and are located in A compartment regions with a significant increase in eigenvector 1 (stronger A compartment score) upon ATRA treatment. 2. Genes that were downregulated in the BKO + ATRA condition compared to WT + ATRA and genes located in B compartment regions with significant decreases in eigenvector 1 in BKO + ATRA vs WT + ATRA (Table 1). This set of genes in ATRA treated cells are downregulated in the BKO vs WT genotype and are located in B compartment regions with decreases in eigenvector 1 (stronger B compartment score) in BKO vs WT cells.

**Table 1:** Differential compartment and gene expression comparison

Comparison	direction	de_gene _count	diff_comp_gene _count	both_gene _count	p-value_ hyper
WT_ATRAvsWT_Ctrl	up	673	70	4	0.120
WT_ATRAvsWT_Ctrl	down	328	900	8	0.907
TOP2BKO_ATRAvsTOP2BKO_Ctrl	up	486	243	10	0.021
TOP2BKO_ATRAvsTOP2BKO_Ctrl	down	270	793	9	0.487
TOP2BKO_CtrlvsWT_Ctrl	up	673	63	3	0.240
TOP2BKO_CtrlvsWT_Ctrl	down	974	42	4	0.080
TOP2BKO_ATRAvsWT_ATRA	up	619	222	5	0.646
TOP2BKO_ATRAvsWT_ATRA	down	1202	239	19	0.022

As the overlap between the differentially expressed genes and the differential compartment regions was small, we next analyzed the distribution of eigenvector 1 in all bins overlapping differentially expressed genes (Fig. 5C), to determine if there was a trend across many DE genes that may have been missed by the differential compartment analysis. We found overall that the eigenvector 1 values were decreased in the ATRA treatment compared to the control for both cell lines, and that the range of values was reduced in the TOP2B KO genotype. While the eigenvector 1 values did not change in the direction expected for all gene classes, for the class of genes whose expression levels were increased in the WT only with ATRA treatment, we found that there was a trend towards decreased eigenvector 1 values in the BKO ATRA compared to BKO control, while the WT eigenvector 1 distributions remained similar. This suggests that TOP2B is involved in maintaining the A compartment state upon ATRA treatment at these loci. In addition, genes that were upregulated in both genotypes upon ATRA treatment also showed increased eigenvector 1 values in both WT and BKO cells, and genes downregulated in both genotypes showed decreased eigenvector 1 values in both WT and BKO cells.

### Examples of genes concordantly compartmentalized and expressed between genotypes or treatments

In Figs S1 and S2, we show examples of the Hi-C maps of two differentially regulated genes between WT and BKO ATRA treated cells which fall within differential compartment regions. VAT1L is expressed in the brain, eye, and other tissues (https://www.proteinatlas.org/ENSG00000171724-VAT1L), and has been detected in Horizontal cells (neuronal), Inhibitory Neurons (neuronal) and glial cells by single-cell RNA-seq (https://www.proteinatlas.org/ENSG00000171724-VAT1L/single+cell+type). In WT SH-SY5Y cells *VAT1L* lies in a small A compartment upon ATRA treatment, however in the BKO cells this small A compartment is lost, and *VAT1L* is not expressed (Fig. S1). In contrast, FGFR2 is a gene that is more highly expressed and has a stronger A compartment score in the BKO cell line than in the WT with ATRA treatment (Fig. S2). FGFR2 has roles in fibroblast growth, wound repair, and angiogenesis (https://www.genecards.org/cgi-bin/carddisp.pl?gene=FGFR2#publications), and in the brain is mainly expressed in astrocytes and oligodendrocytes, which are glial cells, rather than in neuronal cells (https://www.proteinatlas.org/ENSG00000066468-FGFR2/single+cell+type). In contrast to the two examples above, *CYP26B1* and *NTRK2* are two genes whose expression is robustly induced by ATRA in SH-SY5Y cells. *CYP26B1* is involved in retinoic acid metabolism and is rapidly induced by ATRA in both cell lines, although to a somewhat lesser degree in BKO cells (Khazeem et al. 2022). *CYP26B1* resides in a small B compartment in control cells that is lost upon ATRA treatment in both WT and BKO cells, consistent with the *CYP26B1* gene becoming expressed (Fig. S3). Similarly, *NTRK2* is an ATRA induced gene required for normal neural development. In control conditions it is expressed at a considerably lower level in BKO than WT cells, but the fold increase in expression with ATRA is the same in both cell lines. Like *CYP26B1*, *NTRK2* resides in a B domain in untreated cells and matching the expression pattern, this B domain is lost upon ATRA treatment (Fig. S4). For both of these genes, ATRA treatment resulted in compartment change, and this was not dramatically affected by loss of TOP2B.

To summarize, we have previously reported a large number of gene expression changes comparing WT and BKO SH-SY5Y cells, and upon ATRA treatment of these cells (Khazeem et al. 2022). In the present study we show that there are global changes in chromosome organization upon ATRA treatment which are less pronounced in the BKO cell line, however most of these changes are not specifically localized near differentially expressed genes. We saw no evidence for changes in cohesin loops or TADs in any of the comparisons. However, lack of TOP2B did affect compartment strength changes that occur upon ATRA treatment and attenuated the shift away from a Rabl-like conformation observed in control cells upon ATRA treatment. Overall, we find that both A and B compartment strength is affected by TOP2B deletion upon ATRA differentiation compared to the WT cells upon ATRA differentiation, however only a subset of genes appear to be directly affected by this change in compartmentalization at the 24 h timepoint. The relatively subtle effect of TOP2B gene inactivation on the 3D architecture of the genome, and particularly the lack of change in cohesin loops and TADs leads to the conclusion that the observed transcriptional requirements for TOP2B are not connected to these long-range chromatin configurations. This leaves open the question of what does underly the requirement for TOP2B for rapid induction of gene activity and in certain developmental programs. As discussed elsewhere (Cowell et al. 2023), this might involve TOP2B-mediated DSBs at gene regulatory regions. A second unanswered question surrounds the common association of TOP2B with CTCF genomic binding sites, with one possibility being the modulation of transcription-linked supercoiling within topologically constrained TADs rather than a role in the stability or dynamics of the TADs themselves.

## Methods

### Cell culture

Wildtype SH-SY5Y and TOP2B null clone BKO98 (Khazeem et al. 2022) clones were maintained in a 50:50 mixture of Minimum Essential Medium (MEM) and F12 (Hams) medium (Gibco by Life Technologies, Invitrogen, UK) with 10% v/v fetal bovine serum (FBS) (Gibco by Life Technologies, Invitrogen, UK) and 1% v/v Penicillin and Streptomycin solution (10,000 units/ml penicillin, 10,000 mg/ml streptomycin, Gibco by Life Technologies, Invitrogen, UK). Cells were passaged using 0.5mM EDTA in PBS; after 2–4 min incubation at room temperature cells were dislodged by knocking the side of the flask, and then triturating using a 10ml serological pipette to encourage suspension into single cells. Typically, cells were passaged at a 1:4 ratio.

### Retinoic acid treatment

WT and BKO98 SH-SY5Y cells were plated into 20cm culture dishes in medium containing 10µM ATRA in ethanol or the same volume of ethanol (0.1% v/v final concentration of ethanol). Cells were cultured for 5 days and medium was changed on days 2 and 4 to maintain ATRA concentration.

### Cell Cycle profiling

Due to the need to grow the cells and treat them with ATRA in adherent monolayer culture and their tendency to clump when removed from the substrate, cell cycle profiles were assessed by quantitative DAPI fluorescence microscopy essentially as per Roukos et al. (Roukos et al. 2015). Cells were plated onto round coverslips in wells of 6 well plates in medium containing 10µM ATRA in ethanol or the same volume of ethanol (0.1% v/v final concentration of ethanol). Cells were cultured for 7 days and medium was changed on days 2 and 4 to maintain ATRA concentration. Cells were then fixed in 4% PFA in PBS for 10 min at RT and coverslips were then washed with PBS before permeabilization with 0.3% Triton X-100 in PBS for 5 min. Coverslips were then washed with PBS and cells were stained for 20 min in the dark with DAPI (0.2μg/ml DAPI in PBS). Coverslips were mounted onto slides using Vectashield mounting medium. Quantitative fluorescence microscopy for cell cycle analysis was carried out using an Olympus IX-81 epifluorescence microscope (10X objective) fitted with an Orca-AG camera (Hamamatsu, no binning) and suitable narrow band filter. Image analysis was performed with Volocity (Perkin-Elmer). Further analysis and graph plotting was carried out using R as modified from Roukos et al. (Roukos et al. 2015).

### Immunofluorescence

For examination of nucleolar properties cells were grown and fixed as above (Cell cycle profiling), but before counterstaining with DAPI coverslips were processed for immunofluorescence with anti-fibrillarin ab18380 (Abcam) using the immunofluorescent protocol described in Cowell et al. (Cowell et al. 2019). For fibrillarin staining, images were captured using a 40X objective and extended focus images prepared from z-stacks using Volocity are presented.

### RAD21 chromatin association

To quantify the relative amount and chromatin association of cohesin (RAD21) WT and BKO98 cells were spread on glass slides as a thin layer in suspension in agarose, as per the TARDIS protocol described by Cowell and Austin (Cowell and Austin 2018). Embedded cells were then either fixed with 4% PFA and then permeabilized to quantify total RAD21 or TOP2A, or extracted in CSK1 buffer (Poonperm et al. 2017) (10mM PIPES, pH 6.8, 10% glycerol, 3 mM MgCl2 100 mM NaCl, 1 mM DTT, 0.3% Triton, protease inhibitor cocktail) for 30 min on ice and then fixed, to measure chromatin bound RAD21 or TOP2A. Immunofluorescence was carried out using anti-RAD21 ab154769 (Abcam). Images were captured as above using a 10X objective and analysis for quantitative immunofluorescence was performed using Volocity (Perkin-Elmer) and Prism 4.0 (Graphpad).

### Western blotting

Whole cell extracts were prepared as described by Mirski (Mirski et al. 1993). Samples (25µg) were separated on Biorad minigels and blotted onto nitrocellulose. Antibody incubations and detection were by standard protocols using ECL detection employing a Li-cor C-digit detector.

### Hi-C

Hi-C was performed using the Hi-C 2.0 protocol (Belaghzal et al. 2017) with the addition of DSG crosslinking. Approximately 5 × 10^6^ cells were fixed in 1% formaldehyde (Fisher, BP531-25) diluted in serum-free media, as previously described. Formaldehyde fixation was quenched with 0.125 M Glycine for 5 min at room temp, and 15 min on ice. Cells were scraped from the plate and washed after formaldehyde fixation with PBS and were then resuspended with the 3 mM DSG crosslinking solution (stock solution 300 mM in DMSO, diluted 1:100 in PBS for fixation), and were incubated by rotation at room temp for 40 min. DSG crosslinking was quenched with 0.4 M Glycine, incubated for 5 min at room temp, and then spun down at 2000xg for 15 min. Cells were washed with 10 ml cold 0.05% BSA in PBS to reduce clump formation and were spun again at 2000xg for 15 min. Supernatant was removed and cells were flash frozen in liquid N2 and stored at −80 degrees C.

Flash-frozen cross-linked cells were thawed on ice for Hi-C, and were then lysed and digested with DpnII (NEB, R0543M) at 37 degrees C overnight, following the Hi-C 2.0 protocol (Belaghzal et al. 2017). The overhanging DNA ends were filled in using biotin-14-dATP (LifeTech, 19,524,016) at 23 degrees C for four hours, and ligated with T4 DNA ligase (Life Technologies, 15,224,090) at 16 degrees C for four hours. Chromatin was then treated with proteinase K (ThermoFisher, 25,530,031) at 65 degrees C overnight to remove all cross-linked proteins. Ligation products were purified by phenol:chloroform extraction with ethanol precipitation, fragmented by sonication, and size-selected using SPRI beads to retain fragments of 100–350 bp. Next, we performed end repair and then selectively purified biotin-tagged DNA using streptavidin coated beads (DYNAL™ MyOne™ Dynabeads™ Streptavidin C1, Invitrogen 65,001). A-tailing and Illumina TruSeq adapter ligation (Illumina, 20,015,964) were performed on the bead-bound ligation products, and samples were amplified using the TruSeq Nano DNA Sample Prep kit (Illumina, 20,015,964). PCR primers were removed using SPRI beads (1.1 × ratio) before sequencing the final Hi-C libraries using PE50 bases on an Illumina HiSeq 4000.

### Hi-C analysis

#### Read mapping

Hi-C data processing was performed as previously described (Abramo et al. 2019) to the hg38 human reference genome using the nextflow based distiller-nf pipeline. The number of valid pairs was normalized within each replicate using cooltools (v0.5.1) random-sample with the –exact argument on 1 kb resolution.cool files (Abdennur et al. 2024). MultiQC was used for quality control of the Hi-C libraries (Ewels et al. 2016). Read normalized valid pairs were binned into.mcool formatted contact matrices using the python package cooler (v0.8.11), and were iteratively balanced (Abdennur and Mirny 2020, Abdennur et al. 2021). Hi-C analysis was performed using Hi-C specific python packages such as cooler (v0.8.11), cooltools (v0.5.1), bioframe (v0.3.3) and pairtools (v0.3.0) (Goloborodko et al. 2019, Abdennur et al. 2021, 2022). Other python packages used included pandas (v1.4.2), numpy (v1.22.3), scipy (v1.8.0), scikit-image (v0.19.2), seaborn (v0.11.2) and matplotlib (v3.5.2) (Hunter 2007, McKinney, 2010, van der Walt et al. 2014, Harris et al. 2020, Virtanen et al. 2020, Reback et al. 2021). Only chromosomes without major translocations were used for most analysis (chr1-chr6, chr9-chr14, chr16, chr18-chr21).

#### Distance decay

Contact frequency (*P*) as a function of genomic distance (*s*) and the derivative (slope) of this *P(s)* curve were calculated using cooltools compute-expected followed by cooltools logbinned-expected using only intrachromosomal reads from the read normalized cooler files, binned at 1 Kb.

#### TAD insulation

Insulation between topologically associating domains was calculated on 10 kb binned.cool files using the cooltools API function calculate_insulation_score, with a 250 kb diamond sliding window (Abdennur et al. 2022). Domain boundaries were found by locating minima in each profile, and thresholding using skimage threshold_otsu (van der Walt et al. 2014). Boundaries were then filtered to remove boundaries that overlapped with changes in compartment type, to analyze TAD only boundary strength. Aggregate insulation plots were made by plotting the average insulation in 500 kb windows around all called TAD boundaries in the WT Control Hi-C library (Hunter 2007).

#### Compartment analysis

Active and inactive compartment regions were identified using eigenvector decomposition on 250 kb binned Hi-C.mcool files using cooltools, as previously described (Imakaev et al. 2012, Abramo et al. 2019; Abdennur et al. 2022). The eigenvector was phased such that positive values corresponded to the more gene-dense regions of the genome (the ‘A’, or active compartment), while negative values corresponded to the gene-poor regions of the genome (the ‘B’ or inactive compartment). To measure the strength of compartmentalization, we used cooltools saddleplot analysis on observed/expected Hi-C data, as previously described, where the expected matrix corresponds to the average distance decay (Imakaev et al. 2012; Abramo et al. 2019; Abdennur et al. 2022). Observed/expected matrix bins within or between chromosomes were sorted and aggregated into 50 bins according to their eigenvalue. Strength of compartmentalization for each distance was calculated as the ratio of AA/AB or BB/AB interactions, where AA is the average of the corner 10 bins with positive, positive eigenvector values, BB is the average of the 10 corner bins with negative, negative eigenvector values, and AB is the average of the 10 bins in the corner with positive, negative eigenvector values.

#### Differential gene expression comparison

Differential compartment bins were called and compared to differentially expressed genes using dcHiC-v1 (Chakraborty et al. 2022), with E1 values calculated using cooltools. The fraction of differential compartment bins was calculated compared to all bins in the genome with non-NA compartment values (FDR < 0.01). A hypergeometric distribution test was used to calculate the p value of as many or more genes than observed overlapping between the RNA-seq differential genes and the genes in differential compartment domains using scipy.phyper. The eigenvector values for all bins overlapping differentially expressed genes in different classes were plotted as a boxplot using seaborn. Correlated changes in gene expression and compartment eigenvector value in the WT ATRA differentiation were identified, and the E1 for these regions was plotted for both WT and Top2BKO cell lines.

#### Average inter and intra-chromosomal interactions

Scaled inter and intra-chromosomal interaction pileups were visualized using Cooltools version 0.5.4 with the following edits: in the cooltools saddle.py script in api folder, functions def _make_cis_obsexp_fetcher and _make_trans_obsexp_fetcher were edited to return only observed matrix instead of obs/expected (see saddle_obs.py script on Gitlab). Scaled centromere to telomere tracks were generated for chromosomes 1–22 at 100kb bin size, by assigning values from 0 to 1 for the p arm, and 1 to 2 for the q arm with 1 representing the centromeric region and 0 and 2 the telomeres (CentTeltrack.py). These scaled centromere to telomere tracks were used to pileup the observed Hi-C interactions across all chromosomes either in cis (intra-chromosomal) or in trans (inter-chromosomal). For inter-chromosomal pileups, the plot_saddle function was used, while for intra-chromosomal pileups the plot_saddle_flexible function was used. plot_ratio_matrix_withlineplot was used to generate the ratio plots for intra and inter-chromosomal pileup data.

## Supplementary Information

Below is the link to the electronic supplementary material.

Supplementary file1 (PDF 665 KB)

## Data Availability

Hi-C short-read sequencing data have been deposited at GEO and will be publicly available as of the date of publication (GSE252149). All original code is deposited at Zenodo https://zenodo.org/records/15099011 upon. Published datasets used: (SH-SY5Y RNA-seq data, GSE142383).

## References

[CR1] Abdennur N, Mirny LA (2020) Cooler: scalable storage for Hi-C data and other genomically labeled arrays. Bioinformatics 36:311–316 31290943 10.1093/bioinformatics/btz540 PMC8205516

[CR2] Abdennur N, Goloborodko A, Imakaev M, Kerpedjiev P, Fudenberg G, Oullette S, et al (2021) open2c/cooler: v0.8.11. v0.8.11 ed: Zenodo. 10.5281/zenodo.4655850

[CR3] Abdennur N, Fudenberg G, Flyamer I, Galitsyna AA, Goloborodko A, Imakaev M, et al (2022) Bioframe: operations on genomic intervals in pandas Data frames. Bioinform 40(2):btae088. 10.1093/bioinformatics/btae088 10.1093/bioinformatics/btae088 PMC10903647 38402507

[CR4] Abdennur N, Abraham S, Fudenberg G, Flyamer IM, et al. (2024) Cooltools: enabling high resolution Hi-C analysis in Python. PLoS Comput Biol 20(5): e1012067. https://journals.plos.org/ploscompbiol/article?id=10.1371/journal.pcbi.1012067 10.1371/journal.pcbi.1012067 PMC11098495 38709825

[CR5] Abramo K, Valton AL, Venev SV, Ozadam H, Fox AN, Dekker J (2019) A chromosome folding intermediate at the condensin-to-cohesin transition during telophase. Nat Cell Biol 21:1393–1402 31685986 10.1038/s41556-019-0406-2 PMC6858582

[CR6] Austin CA, Lee KC, Swan RL, Khazeem MM, Manville CM, Cridland P, et al (2018) TOP2B: the first thirty years. Int J Mol Sci 19. 10.3390/ijms 10.3390/ijms19092765 PMC6163646 30223465

[CR7] Baxter J (2024) Entangling and disentangling mitotic chromosomes. Mol Cell 84:1398–1400 38640891 10.1016/j.molcel.2024.03.025

[CR8] Belaghzal H, Dekker J, Gibcus JH (2017) Hi-C 2.0: An optimized Hi-C procedure for high-resolution genome-wide mapping of chromosome conformation. Methods. 123:56–65 10.1016/j.ymeth.2017.04.004 PMC5522765 28435001

[CR9] Bell N, Hann V, Redfern CPF, Cheek TR (2013) Store-operated Ca2 + entry in proliferating and retinoic acid-differentiated N- and S-type neuroblastoma cells. Biochimica et Biophysica Acta (BBA) – Mol Cell Res 1833:643–51 10.1016/j.bbamcr.2012.11.025 PMC3776921 23220046

[CR10] Brahmachari S, Marko JF (2019) Chromosome disentanglement driven via optimal compaction of loop-extruded brush structures. Proc Natl Acad Sci U S A 116:24956–24965 31757850 10.1073/pnas.1906355116 PMC6911191

[CR11] Broderick L, Yost S, Li D, McGeough MD, Booshehri LM, Guaderrama M et al (2019) Mutations in topoisomerase IIbeta result in a B cell immunodeficiency. Nat Commun 10:3644 31409799 10.1038/s41467-019-11570-6 PMC6692411

[CR12] Bunch H, Lawney BP, Lin Y-F, Asaithamby A, Murshid A, Wang YE, et al. (2015) Transcriptional elongation requires DNA break-induced signalling. Nat Commun 6. 10.1038/ncomms10191 10.1038/ncomms10191 PMC4703865 26671524

[CR13] Canela A, Maman Y, Jung S, Wong N, Callen E, Day A et al (2017) Genome Organization Drives Chromosome Fragility. Cell 170(507–21):e18 10.1016/j.cell.2017.06.034 PMC6133249 28735753

[CR14] Chakraborty A, Wang JG, Ay F (2022) dcHiC detects differential compartments across multiple Hi-C datasets. Nat Commun 13:6827 36369226 10.1038/s41467-022-34626-6 PMC9652325

[CR15] Cowell IG, Austin CA (2018) Visualization and Quantification of Topoisomerase-DNA Covalent Complexes Using the Trapped in Agarose Immunostaining (TARDIS) Assay. Methods Mol Biol 1703:301–316 29177750 10.1007/978-1-4939-7459-7_21

[CR16] Cowell IG, Ling EM, Swan RL, Brooks MLW, Austin CA (2019) The Deubiquitinating Enzyme Inhibitor PR-619 is a Potent DNA Topoisomerase II Poison. Mol Pharmacol 96:562–572 31515282 10.1124/mol.119.117390 PMC6776009

[CR17] Cowell IG, Casement JW, Austin CA (2023) To Break or Not to Break: The Role of TOP2B in Transcription. In: Deweese JE, Osheroff N (eds) Advances in Structure. MDPI, Function and Molecular Targeting of DNA Topoisomerases, pp 4–18 10.3390/ijms241914806 PMC10573011 37834253

[CR18] Erdel F, Rippe K (2018) Formation of Chromatin Subcompartments by Phase Separation. Biophys J 114:2262–2270 29628210 10.1016/j.bpj.2018.03.011 PMC6129460

[CR19] Ewels P, Magnusson M, Lundin S, Kaller M (2016) MultiQC: summarize analysis results for multiple tools and samples in a single report. Bioinformatics 32:3047–3048 27312411 10.1093/bioinformatics/btw354 PMC5039924

[CR20] Fudenberg G, Imakaev M, Lu C, Goloborodko A, Abdennur N, Mirny LA (2016) Formation of Chromosomal Domains by Loop Extrusion. Cell Rep 15:2038–2049 27210764 10.1016/j.celrep.2016.04.085 PMC4889513

[CR21] Goloborodko A, Abdennur N, Venev S (2019) hbbrandao, gfudenberg. mirnylab/pairtools: v0.2.2. v0.2.2 ed: Zenodo. 10.5281/zenodo.1490831

[CR22] Haffner MC, Aryee MJ, Toubaji A, Esopi DM, Albadine R, Gurel B et al (2010) Androgen-induced TOP2B-mediated double-strand breaks and prostate cancer gene rearrangements. Nat Genet 42:668–675 20601956 10.1038/ng.613 PMC3157086

[CR23] Harkin LF, Gerrelli D, Gold Diaz DC, Santos C, Alzu’bi A, Austin CA et al (2016) Distinct expression patterns for type II topoisomerases IIA and IIB in the early foetal human telencephalon. J Anat 228:452–463 26612825 10.1111/joa.12416 PMC4832326

[CR24] Harris CR, Millman KJ, van der Walt SJ, Gommers R, Virtanen P, Cournapeau D et al (2020) Array programming with NumPy. Nature 585:357–362 32939066 10.1038/s41586-020-2649-2 PMC7759461

[CR25] Hildebrand EM, Dekker J (2020) Mechanisms and Functions of Chromosome Compartmentalization. Trends Biochem Sci 45:385–396 32311333 10.1016/j.tibs.2020.01.002 PMC7275117

[CR26] Hildebrand EM, Polovnikov K, Dekker B, Liu Y, Lafontaine DL, Fox AN et al (2024) Mitotic chromosomes are self-entangled and disentangle through a topoisomerase-II-dependent two-stage exit from mitosis. Mol Cell 84:1422–41.e14 38521067 10.1016/j.molcel.2024.02.025 PMC11756355

[CR27] Hsieh THS, Cattoglio C, Slobodyanyuk E, Hansen AS, Darzacq X, Tjian R (2022) Enhancer–promoter interactions and transcription are largely maintained upon acute loss of CTCF, cohesin, WAPL or YY1. Nat Genet 54:1919–1932 36471071 10.1038/s41588-022-01223-8 PMC9729117

[CR28] Hung TC, Kingsley DM, Boettiger AN (2024) Boundary stacking interactions enable cross-TAD enhancer-promoter communication during limb development. Nat Genet 56:306–314 38238628 10.1038/s41588-023-01641-2 PMC13730118

[CR29] Hunter JD (2007) Matplotlib: A 2D Graphics Environment. Comput Sci Eng 9:90–95

[CR30] Imakaev M, Fudenberg G, McCord RP, Naumova N, Goloborodko A, Lajoie BR et al (2012) Iterative correction of Hi-C data reveals hallmarks of chromosome organization. Nat Methods 9:999–1003 22941365 10.1038/nmeth.2148 PMC3816492

[CR31] Ju BG, Lunyak VV, Perissi V, Garcia-Bassets I, Rose DW, Glass CK et al (2006) A topoisomerase IIβ-mediated dsDNA break required for regulated transcription. Science 312:1798–1802 16794079 10.1126/science.1127196

[CR32] Khazeem MM, Casement JW, Schlossmacher G, Kenneth NS, Sumbung NK, Chan JYT et al (2022) TOP2B is required to maintain the adrenergic neural phenotype and for ATRA-Induced differentiation of SH-SY5Y Neuroblastoma Cells. Mol Neurobiol 59:5987–6008 35831557 10.1007/s12035-022-02949-6 PMC9463316

[CR33] Kouzine F, Baranello L, Levens D (2018) The use of psoralen photobinding to study transcription-induced supercoiling. Methods Mol Biol 1703:95–108 29177736 10.1007/978-1-4939-7459-7_7 PMC7433354

[CR34] Kovalevich J, Langford D (2013) Considerations for the use of SH-SY5Y neuroblastoma cells in neurobiology. Methods Mol Biol 1078:9–21 23975817 10.1007/978-1-62703-640-5_2 PMC5127451

[CR35] Lieberman-Aiden E, van Berkum NL, Williams L, Imakaev M, Ragoczy T, Telling A et al (2009) Comprehensive mapping of long-range interactions reveals folding principles of the human genome. Science 326:289–293 19815776 10.1126/science.1181369 PMC2858594

[CR36] Liu LF, Wang JC (1987) Supercoiling of the DNA template during transcription. Proc Natl Acad Sci U S A 84:7024–7027 2823250 10.1073/pnas.84.20.7024 PMC299221

[CR37] Ma J, Wang MD (2016) DNA Supercoiling during Transcription. Biophys Rev 8:75–87 28275417 10.1007/s12551-016-0215-9 PMC5338639

[CR38] Madabhushi R, Gao F, Pfenning AR, Pan L, Yamakawa S, Seo J et al (2015) Activity-induced DNA breaks govern the expression of neuronal early-response genes. Cell 161:1592–1605 26052046 10.1016/j.cell.2015.05.032 PMC4886855

[CR39] Manville CM, Smith K, Sondka Z, Rance H, Cockell S, Cowell IG et al (2015) Genome-wide ChIP-seq analysis of human TOP2B occupancy in MCF7 breast cancer epithelial cells. Biol Open 4:1436–1447 26459242 10.1242/bio.014308 PMC4728365

[CR40] McKinney W (2010) Data structures for statistical computing in python. In: Walt Svd, Millman J (eds) p 56–61. https://proceedings.scipy.org/articles/Majora-92bf1922-00a

[CR41] Mirski SE, Evans CD, Almquist KC, Slovak ML, Cole SP (1993) Altered topoisomerase II alpha in a drug-resistant small cell lung cancer cell line selected in VP-16. Cancer Res 53:4866–4873 8104687

[CR42] Naughton C, Avlonitis N, Corless S, Prendergast JG, Mati IK, Eijk PP et al (2013) Transcription forms and remodels supercoiling domains unfolding large-scale chromatin structures. Nat Struct Mol Biol 20:387–395 23416946 10.1038/nsmb.2509 PMC3689368

[CR43] Naumova N, Imakaev M, Fudenberg G, Zhan Y, Lajoie BR, Mirny LA et al (2013) Organization of the mitotic chromosome. Science 342:948–953 24200812 10.1126/science.1236083 PMC4040465

[CR44] Nora EP, Goloborodko A, Valton A-L, Gibcus JH, Uebersohn A, Abdennur N et al (2017) Targeted degradation of CTCF decouples local insulation of chromosome domains from genomic compartmentalization. Cell 169:930–44.e22 28525758 10.1016/j.cell.2017.05.004 PMC5538188

[CR45] Padget K, Pearson AD, Austin CA (2000) Quantitation of DNA topoisomerase IIalpha and beta in human leukaemia cells by immunoblotting. Leukemia 14:1997–2005 11069037 10.1038/sj.leu.2401928

[CR46] Polovnikov K, Slavov B (2023) Topological and nontopological mechanisms of loop formation in chromosomes: effects on the contact probability. Phys Rev E 107:054135 37329090 10.1103/PhysRevE.107.054135

[CR47] Poonperm R, Takata H, Uchiyama S, Fukui K (2017) Interdependency and phosphorylation of KIF4 and condensin I are essential for organization of chromosome scaffold. PLoS ONE 12:e0183298 28817632 10.1371/journal.pone.0183298 PMC5560531

[CR48] Rao SS, Huntley MH, Durand NC, Stamenova EK, Bochkov ID, Robinson JT et al (2014) A 3D map of the human genome at kilobase resolution reveals principles of chromatin looping. Cell 159:1665–1680 25497547 10.1016/j.cell.2014.11.021 PMC5635824

[CR49] Reback J, McKinney W, jbrockmendel, Bossche JVd, Augspurger T, Cloud P, et al (2021) Pandas-dev/pandas: pandas 1.2.4. v1.2.4 ed: Zenodo. 10.5281/zenodo.4681666

[CR50] Roukos V, Pegoraro G, Voss TC, Misteli T (2015) Cell cycle staging of individual cells by fluorescence microscopy. Nat Protoc 10:334–348 25633629 10.1038/nprot.2015.016 PMC6318798

[CR51] Spracklin G, Abdennur N, Imakaev M, Chowdhury N, Pradhan S, Mirny LA et al (2023) Diverse silent chromatin states modulate genome compartmentalization and loop extrusion barriers. Nat Struct Mol Biol 30:38–51 36550219 10.1038/s41594-022-00892-7 PMC9851908

[CR52] Teves SS, Henikoff S (2014) Transcription-generated torsional stress destabilizes nucleosomes. Nat Struct Mol Biol 21:88–94 24317489 10.1038/nsmb.2723 PMC3947361

[CR53] Tiwari VK, Burger L, Nikoletopoulou V, Deogracias R, Thakurela S, Wirbelauer C et al (2012) Target genes of Topoisomerase IIbeta regulate neuronal survival and are defined by their chromatin state. Proc Natl Acad Sci U S A 109:E934–E943 22474351 10.1073/pnas.1119798109 PMC3340998

[CR54] Trotter KW, King HA, Archer TK (2015) Glucocorticoid receptor transcriptional activation via the BRG1-dependent recruitment of TOP2β and Ku70/86. Mol Cell Biol 35:2799–2817 26055322 10.1128/MCB.00230-15 PMC4508321

[CR55] Uuskula-Reimand L, Hou H, Samavarchi-Tehrani P, Rudan MV, Liang M, Medina-Rivera A et al (2016) Topoisomerase II beta interacts with cohesin and CTCF at topological domain borders. Genome Biol 17:182 27582050 10.1186/s13059-016-1043-8 PMC5006368

[CR56] van der Walt S, Schonberger JL, Nunez-Iglesias J, Boulogne F, Warner JD, Yager N et al (2014) scikit-image: image processing in Python. PeerJ 2:e453 25024921 10.7717/peerj.453 PMC4081273

[CR57] Virtanen P, Gommers R, Oliphant TE, Haberland M, Reddy T, Cournapeau D et al (2020) SciPy 1.0: fundamental algorithms for scientific computing in Python. Nat Methods 17:261–72 32015543 10.1038/s41592-019-0686-2 PMC7056644

[CR58] Vos SM, Tretter EM, Schmidt BH, Berger JM (2011) All tangled up: how cells direct, manage and exploit topoisomerase function. Nat Rev Mol Cell Biol 12:827–841 22108601 10.1038/nrm3228 PMC4351964

[CR59] Yao Q, Zhu L, Shi Z, Banerjee S, Chen C (2024) Topoisomerase-modulated genome-wide DNA supercoiling domains colocalize with nuclear compartments and regulate human gene expression. Nat Struct Mol Biol. 32(1):48–61 39152238 10.1038/s41594-024-01377-5

[CR60] Zandvliet DW, Hanby AM, Austin CA, Marsh KL, Clark IB, Wright NA et al (1996) Analysis of foetal expression sites of human type II DNA topoisomerase alpha and beta mRNAs by in situ hybridisation. Biochim Biophys Acta 1307:239–247 8679710 10.1016/0167-4781(96)00063-2

[CR61] Zhang S, Liu X, Bawa-Khalfe T, Lu LS, Lyu YL, Liu LF et al (2012) Identification of the molecular basis of doxorubicin-induced cardiotoxicity. Nat Med 18:1639–1642 23104132 10.1038/nm.2919

[CR62] Zuin J, Roth G, Zhan Y, Cramard J, Redolfi J, Piskadlo E et al (2022) Nonlinear control of transcription through enhancer–promoter interactions. Nature 604:571–577 35418676 10.1038/s41586-022-04570-y PMC9021019

