# Supplementary material for: TOP2B is required for compartment strength changes upon retinoic acid treatment in SH-SY5Y cells
Source: Chromosome Res. 2025 Apr 4;33(1):5. doi: 10.1007/s10577-025-09764-4 (PMC11971153; doi:10.1007/s10577-025-09764-4)
Supplement: Supplementary file 1 — Supplementary file1 (PDF 665 KB) [file 10577_2025_9764_MOESM1_ESM.pdf]

Supplementary Figures

Figure S1. Example locus *VAT1L*. Top – Hi-C interaction map and Eigenvector 1 plot of a ~6Mb region of Chr16 containing the *VAT1L* locus from WT control, WT ATRA-treated, BKO control and BKO ATRA-treated cells (light blue: WT Ctrl, dark blue: WT ATRA, light green: BKO Ctrl, dark green: BKO ATRA). Middle – zoomed-in E1 plot containing the *VAT1L* gene (red: A compartment, blue: B compartment). Bottom Log2 fold change RNA-seq values for VAT1L in indicated comparisons.

Figure S2. Example locus *FGFR2*. Top – Hi-C interaction map and Eigenvector 1 plot of a ~4Mb region of Chr10 containing the *FGFR2* locus, from WT control, WT ATRA-treated, BKO control and BKO ATRA-treated cells. (light blue: WT Ctrl, dark blue: WT ATRA, light green: BKO Ctrl, dark green: BKO ATRA). Middle – zoomed-in E1 plot containing the *FGFR2* gene (red: A compartment, blue: B compartment). Bottom Log2 fold change RNA-seq values for FGFR2 in indicated comparisons.

Figure S3. Example locus *CYP26B1*. Top – Hi-C interaction map and Eigenvector 1 plot of a ~5Mb region of Chr10 containing the *CYP26B1* locus, from WT control, WT ATRA-treated, BKO control and BKO ATRA-treated cells (light blue: WT Ctrl, dark blue: WT ATRA, light green: BKO Ctrl, dark green: BKO ATRA). Middle – zoomed-in E1 plot containing the *CYP26B1* gene (red: A compartment, blue: B compartment). Bottom Log2 fold change RNA-seq values for CYP26B1 in indicated comparisons.

Figure S4. Example locus *NTRK2*. Top – Hi-C interaction map and Eigenvector 1 plot of a ~5Mb region of Chr10 containing the *NTRK2* locus, from WT control, WT ATRA-treated, BKO control and BKO ATRA-treated cells (light blue: WT Ctrl, dark blue: WT ATRA, light green: BKO Ctrl, dark green: BKO ATRA). Middle – zoomed-in E1 plot containing the *NTRK2* gene (red: A compartment, blue: B compartment). Bottom Log2 fold change RNA-seq values for NTRK2 in indicated comparisons.

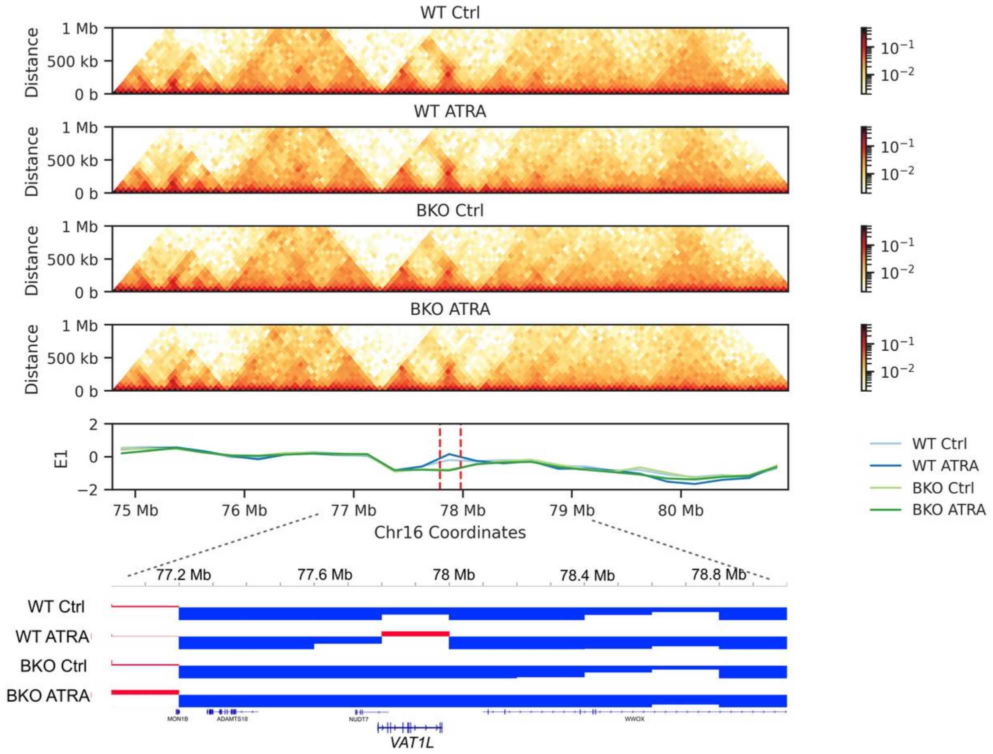

Symbol	WT Con v WT ATRA	BKO Con v BKO ATRA	WT Con v BKO con	WT ATRA v BKO ATRA	WT Con v WT 9cRA 7d
VAT1L	NS	NS	-2.39	-2.49	2.45

Figure S1

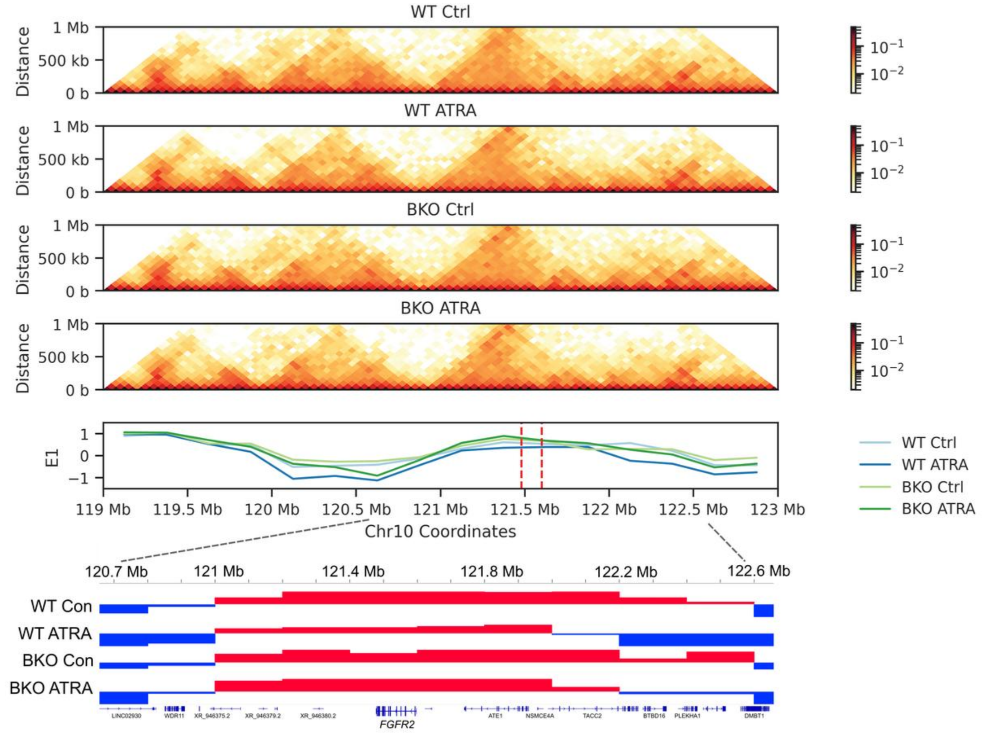

Symbol	WT Con v WT ATRA	BKO Con v BKO ATRA	WT Con v BKO con	WT ATRA v BKO ATRA	WT Con v WT 9cRA 7d
FGFR2	-1.56	NS	0.62	1.69	-0.87

Figure S2

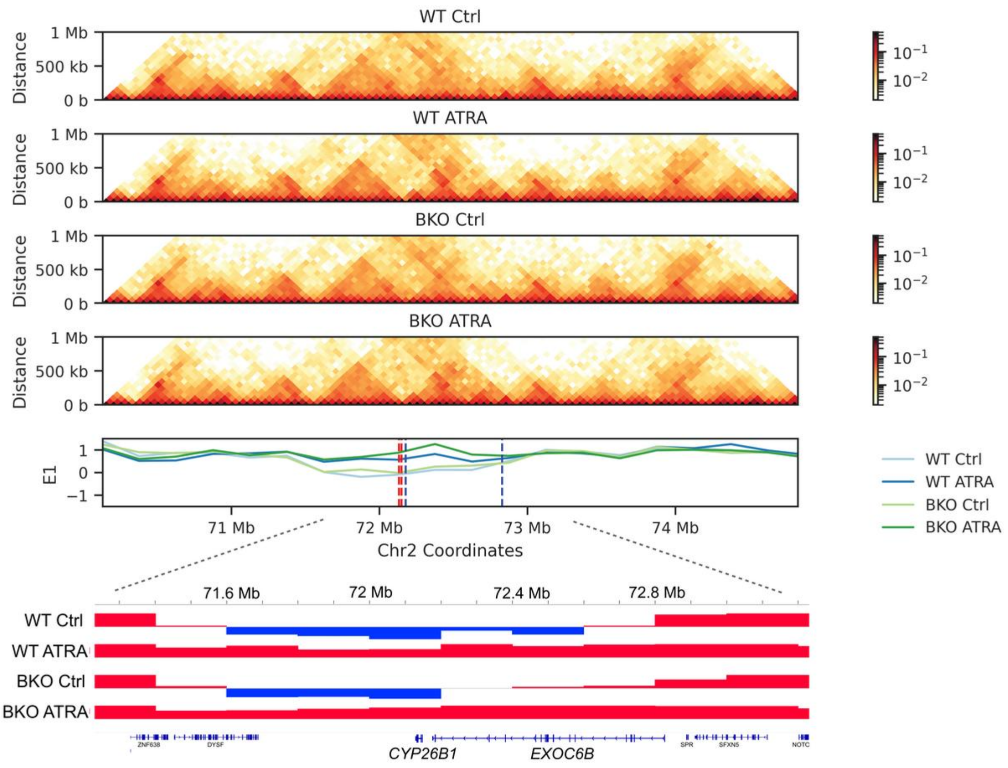

Symbol	WT Con v WT ATRA	BKO Con v BKO ATRA	WT Con v BKO con	WT ATRA v BKO ATRA	WT Con v WT 9cRA 7d
CYP26B1	15.00	11.82	NS	-2.37	NA
EXOC6B	1.80	0.89	NS	-0.77	1.8

Figure S3

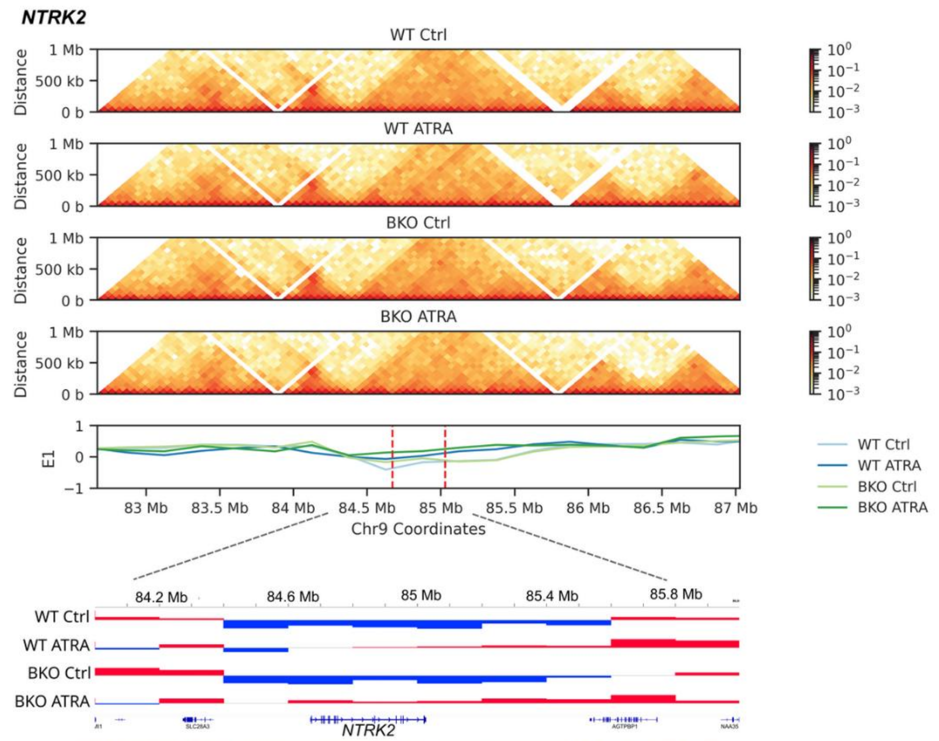

Symbol	WT Con v WT ATRA	BKO Con v BKO ATRA	WT Con v BKO con	WT ATRA v BKO ATRA	WT Con v WT 9cRA 7d
NTRK2	7.67	7.27	-3.23	-3.63	7.24

Figure S4
